# Ocean acidification increases the accumulation of toxic phenolic compounds across trophic levels

**DOI:** 10.1038/ncomms9714

**Published:** 2015-10-27

**Authors:** Peng Jin, Tifeng Wang, Nana Liu, Sam Dupont, John Beardall, Philip W. Boyd, Ulf Riebesell, Kunshan Gao

**Affiliations:** 1 State Key Laboratory of Marine Environmental Science, Xiamen University, Xiamen 361005, China; 2 Department of Biological and Environmental Sciences, University of Gothenburg, 566 Kristineberg, Fiskebäckskil 45178, Sweden; 3 School of Biological Sciences, Monash University, Clayton, Victoria 3800, Australia; 4 Institute for Marine and Antarctic Studies and Antarctic Climate & Ecosystems Cooperative Research Centre, University of Tasmania, Hobart, Tasmania 7005, Australia; 5 GEOMAR Helmholtz Centre for Ocean Research Kiel, Düsternbrooker Weg 20, 24105 Kiel, Germany

## Abstract

Increasing atmospheric CO_2_ concentrations are causing ocean acidification (OA), altering carbonate chemistry with consequences for marine organisms. Here we show that OA increases by 46–212% the production of phenolic compounds in phytoplankton grown under the elevated CO_2_ concentrations projected for the end of this century, compared with the ambient CO_2_ level. At the same time, mitochondrial respiration rate is enhanced under elevated CO_2_ concentrations by 130–160% in a single species or mixed phytoplankton assemblage. When fed with phytoplankton cells grown under OA, zooplankton assemblages have significantly higher phenolic compound content, by about 28–48%. The functional consequences of the increased accumulation of toxic phenolic compounds in primary and secondary producers have the potential to have profound consequences for marine ecosystem and seafood quality, with the possibility that fishery industries could be influenced as a result of progressive ocean changes.

## Abstract (web summary)

Increasing atmospheric CO_2_ concentrations causes ocean acidification, which alters marine chemical environments with unknown consequences for marine ecosystems. Here, Gao *et al*. show that ocean acidification increases levels of phenolic compounds in phytoplankton and zooplankton, implying a food chain impact.

## Graphical abstract

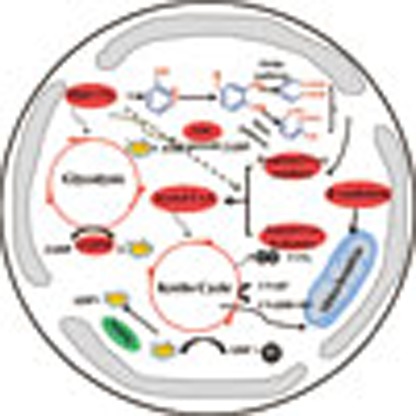

Increasing atmospheric CO_2_ levels are continuing to cause global warming, with increasing oceanic CO_2_ uptake playing an important role in the mediation of the extent of this increase. However, the rapid CO_2_ dissolution into seawater is also causing ocean acidification (OA), which progressively alters marine chemical environments, with consequences for many organisms. While physiological and ecological effects of ocean climate change on primary producers have been examined broadly^1^,^2^, little is known about the molecular aspects and/or metabolic pathways underlying the responses of phytoplankton to OA^3^. Moreover, there are growing concerns about the need to couple the data obtained from monospecific laboratory studies with that from natural communities, as well as under multiple stressor conditions^4^. In addition, the consequences of OA for energy transfer, food quality and the food web remain poorly understood.

To address this issue, we here employ a proteomics approach to investigate the responses of a coccolithophorid, *Emiliania huxleyi* (CCMP 1516) to elevated CO_2_ at the molecular level. On the basis of the findings of the proteomics study, we hypothesize that OA could enhance some metabolic pathways, leading to enhanced production of phenolic compounds. To test this, we measured the levels of phenolic compounds and mitochondrial respiration rates in phytoplankton in monospecific laboratory cultures and in mixed phytoplankton assemblages, grown under different levels of CO_2_. Subsequently, zooplankton assemblages were fed with phytoplankton cells grown under the elevated CO_2_ concentration to examine possible food chain effects. Our results show that OA increases the levels of phenolic compounds in phytoplankton by enhancing β-oxidation, Krebs cycle and mitochondrial respiration, and the accumulated phenolic compounds are transferred to higher trophic levels (zooplankton).

## Results

### Proteomic responses of phytoplankton to OA

**Figure 1:**
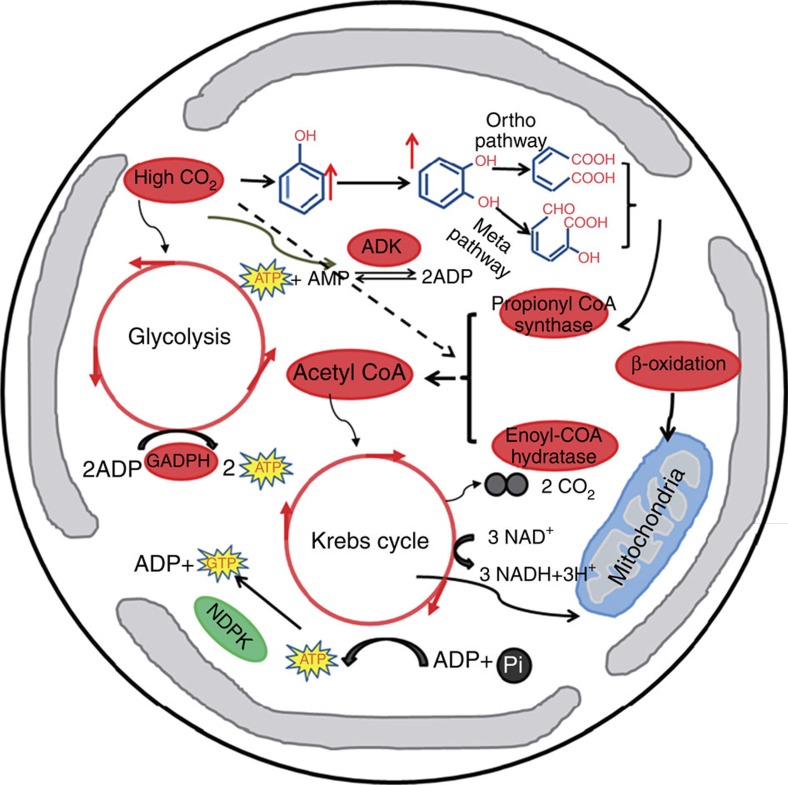
Altered metabolic pathways under ocean acidification. Metabolic pathways in the coccolithophorid *Emiliania huxleyi* altered under ocean acidification (HC, CO_2_ 1,000 μatm; pH_NBS_ 7.81) based on proteomic, physiological and biochemical analyses. More phenolic compounds were biosynthesized, subsequently biodegraded and then metabolized via β-oxidation and Krebs cycle, generating more ATP in the HC-grown cells by mitochondrial respiration. This extra energy can be used to counter the effects of high CO_2_/low pH stress from the environment. Adenylate kinase (ADK) was upregulated, and glycolysis was accelerated in the HC-grown cells. On the other hand, nucleoside diphosphate kinase (NDPK) was downregulated, reflecting the slower loss of ATP loss in the HC-grown cells. The novel pathway including β-oxidation and the Krebs cycle becomes enhanced under ocean acidification to meet the extra energy requirement for maintaining homoeostasis. The red and green symbols represent up- and downregulated proteins or processes, respectively.

When the coccolithophore, *E. huxleyi* (CCMP 1516), was grown monospecifically under low (LC, 395 μatm) pCO_2_ or, high (HC, 1,000 μatm) pCO_2,_ contrasting differences were found in the proteomics (Supplementary Fig. 1). The enzymes methane/phenol/toluene hydroxylase, which plays key roles in phenolic compound biodegradation, propionyl CoA synthase and enoyl CoA hydratase that function in β-oxidation, adenylate kinase (ADK), which is a key enzyme in energy metabolism, and chloroplastic GADPH, were all upregulated in the HC-grown cells (Supplementary Table 1). However, another key enzyme in energy metabolism, nucleoside diphosphate kinase, transferring phosphoryl groups between the adenine and guanine pools (GDP+ATP to GTP+ADP)^5^, was downregulated by about 50% in the HC-grown cells compared with the LC-grown cells (Supplementary Table 1), suggesting that relative ATP loss in cells under HC was slowed down. On the basis of these differentially expressed proteins and their functions, we hypothesized that elevated CO_2_ enhanced the metabolic pathway, described below, leading to enhanced production of phenolic compounds and their metabolism (Fig. 1).

The high CO_2_-induced changes in seawater carbonate chemistry might enhance the cellular production of phenolic compounds, but may also accelerate their biodegradation. As a consequence, enzymes such as phenol hydroxylase would be required for their biodegradation. The resulting products, cis, cis, muconic acid or 2-hydroxymuconic semialdehyde, are further metabolized via β-oxidation and, subsequently, expression of propionyl CoA synthase and enoyl CoA hydratase would be enhanced. The resulting product, acetyl CoA, is a key precursor compound for the Krebs cycle, which functions in oxidizing acetyl CoA to CO_2_ and drives the synthesis of ATP. In such circumstances, the generation of ATP could thus be stimulated in the HC-grown cells (Fig. 1, Supplementary Table 1). Moreover, the enzyme chloroplast GADPH associated with glycolysis was upregulated in the HC-grown cells, indicating that energy demand under the elevated CO_2_ level was higher to operate cellular essential metabolism (Fig. 1, Supplementary Table 1). Together with this, ADK, a key enzyme in energy metabolism, catalysing a reversible transphosphorylation reaction interconverting ADP to ATP and AMP showed significantly higher expression in the HC-grown cells (Fig. 1, Supplementary Table 1). Hence, at least two lines of evidence have to be provided to support the above hypothesis: namely, a higher phenolic compound content and increased mitochondrial respiration rates in *E. huxleyi* grown under high CO_2_ conditions.

### Biochemical and physiological tests

**Figure 2:**
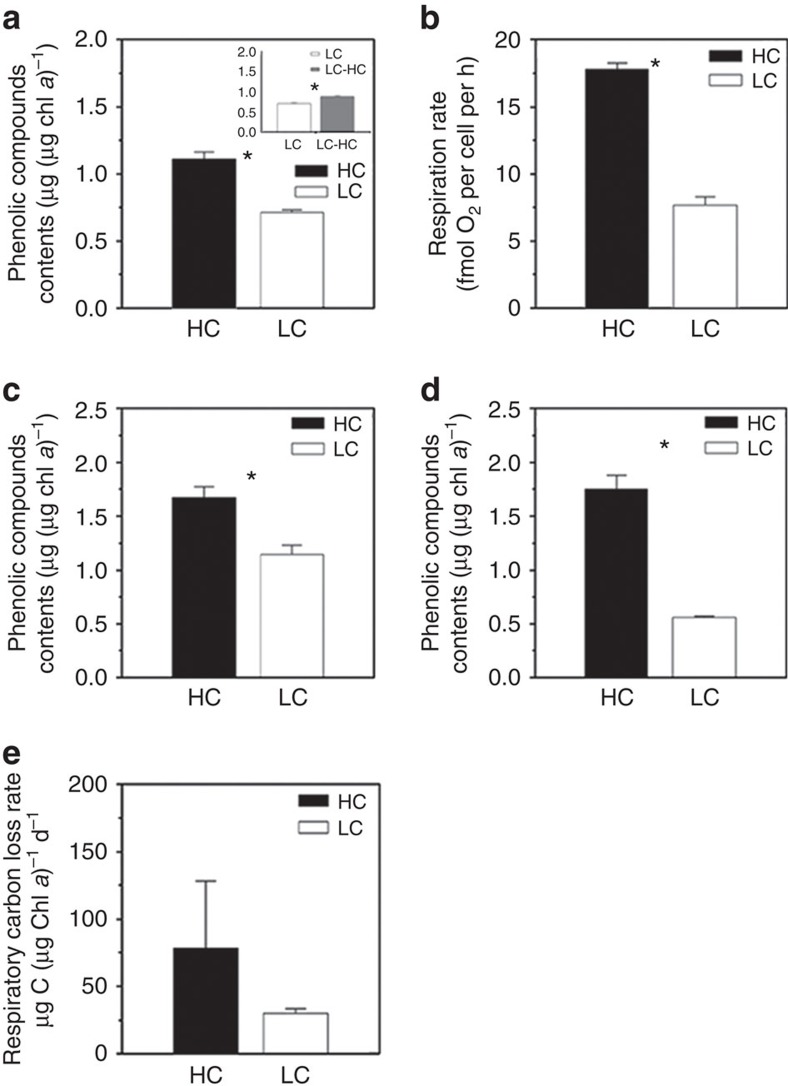
Phenolic compounds contents and respiration rates in phytoplankton. Contents of phenolic compounds and mitochondrial respiration rates under high CO_2_ (HC, 1,000 μatm, pH_NBS_ 7.81) (black column), low pCO_2_ (LC, 395 μatm, pH_NBS_ 8.16; white column) and LC–HC conditions (inset, grey column) in different test systems. (**a**,**b**) Laboratory cultures: contents of phenolic compounds of *Emiliania huxleyi*, μg (μg chlorophyll *a*; chl *a*)^−1^ (**a**); Mitochondrial respiration rates of *E. huxleyi*, fmol O_2_ per cell per h (**b**); (**c**,**d**) Microcosm and mesocosm tests: Phenolic contents of natural phytoplankton assemblages grown in 30 L microcosms, μg (μg chl *a*)^−1^ (**c**); Phenolic contents of phytoplankton assemblages grown in 4,000 L mesocosms, μg (μg chl *a*)^−1^ (**d**); (**e**): Respiratory carbon loss rates of natural phytoplankton assemblages grown in the mesocosms, μg C (μg chl *a*)^−1^ d^−1^). LC–HC (**a**, inset) represents LC-acclimated cells (20 generations) that were transferred to HC conditions for 7 days (∼10 generations). Three independent replicate systems (laboratory cultures, microcosms or mesocosms) were run for each treatment (details for statistics are shown in Supplementary Table 2). Vertical lines represent s.d. of the means. * indicate significance at the *P*<0.05 level.

To test the above hypothesis, we measured the content of phenolic compounds and mitochondrial respiration rates in *E. huxleyi* cells grown under HC and LC conditions. The phenolic compounds were about 56% higher in the HC-acclimated cells than in the LC-acclimated cells (20 generations; analysis of variance (ANOVA) 1, F_1,3_=119.53, *P*<0.001) (Fig. 2a, statistical details in Supplementary Table 2). When the LC-acclimated cells were transferred to HC conditions, the phenolic compound content significantly increased by 24% after a short period of growth (∼10 generations; ANOVA 1, F_1,3_=74.33, *P*=0.01; Fig. 2a, statistical details in Supplementary Table 2). Mitochondrial respiration rates were about 130% higher in the HC-grown cells than in the LC-grown cells (ANOVA 1, F_1,3_=532.66, *P*<0.001; Fig. 2b, statistical details in Supplementary Table 2).

In an attempt to further test our hypothesis at a community level, we conducted a microcosm-level experiment (30 L) with natural coastal phytoplankton assemblages (dominated by the diatoms *Skeletonema costatum* and *Chaetoceros* sp.; details in Supplementary Note 2). We found that phytoplankton assemblages under the high (HC, 1,000 μatm) pCO_2_ treatment showed significantly higher contents of phenolic compounds (by 45.7%) when compared with that of the low pCO_2_ assemblages (ANOVA 2, model: F_5,6_=4.28, *P*=0.048; CO_2_: F_1,6_=18.84, *P*=0.005) (Fig. 2c, statistical details in Supplementary Table 2). During a mesocosm experiment (4,000 L; details in Supplementary Note 2) using The Facility for Ocean Acidification Impacts Study of Xiamen University (24.52°N, 117.18°N, Wuyuan Bay, Xiamen, China, http://mel.xmu.edu.cn/facility.asp?id=33), the phytoplankton assemblages (dominated mainly by diatoms and coccolithophores) also showed elevated, by about 212%, contents of phenolic compounds under high (HC, 1,000 μatm) pCO_2_ (ANOVA 2, model: F_5,12_=10.91, *P*<0.001; CO_2_: F_1,12_=54.48, *P*<0.001; Fig. 2d, statistical details in Supplementary Table 2), compared with that of the low pCO_2_ assemblages. Although the mean values of mitochondrial respiration were higher by 160% in the high CO_2_ mesocosms than in the low CO_2_ treatments, the difference was not significant due to a large variation in the data (ANOVA 1, F_1,3_=2.78, *P*=0.171; Fig. 2e, statistical details in Supplementary Table 2).

### Transfer of phenolics to higher trophic level

**Figure 3:**
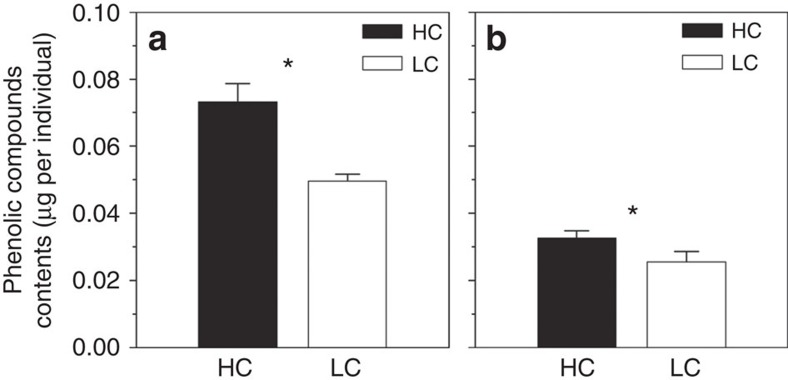
Phenolic compounds contents in zooplankton. Content of phenolic compounds (μg per individual) in zooplankton assemblages (body size >112 μm) that were fed on high CO_2_ (HC, 1,000 μatm, pH_NBS_ 7.81) or low pCO_2_ grown (LC, 395 μatm, pH_NBS_ 8.16) phytoplankton cells collected from the microcosms (**a**, triplicate) or mesocosms (**b**, triplicate). The feeding tests were carried out in *in situ* seawater pre-equilibrated with the high (1,000 μatm) or low pCO_2_ (395 μatm) levels, that is, HC-grown zooplankton fed with HC-grown phytoplankton (black column) or LC-grown zooplankton fed with LC-grown phytoplankton (white column). Details of statistics are shown in Supplementary Table 2. Vertical lines represent the s.d. of the means. * indicates significance at the *P*<0.05 level.

Since biochemical compositions altered by OA in phytoplankton may have profound impacts on trophic energy transfer^6^,^7^, we therefore tested whether zooplankton fed with phytoplankton containing higher levels of phenolic compounds would also have higher concentrations of these toxic compounds in their bodies. When the zooplankton (dominated by calanoid copepods (95%), of which *Acartia pacifica* accounted for up to ∼60%, Supplementary Fig. 2) was fed with HC- and LC-grown phytoplankton cells under the high (1,000 μatm) and low (395 μatm) pCO_2_ levels, higher contents of phenolic compounds were detected in their bodies at elevated pCO_2_, which was true either in the microcosms (47.5%, ANOVA 2, model: F_5,6_=7.19, *P*=0.016; CO_2_: F_1,6_=33.24, *P*=0.001; Fig. 3a) or when fed with phytoplankton cells from the mesocosms (27.8%, ANOVA 2, model: F_5,6_=8.15, *P*=0.012; CO_2_: F_1,6_=29.46, *P*=0.002; Fig. 3b, statistic details in Supplementary Table 2).

## Discussion

On the basis of the outcomes of proteomics in the monospecific study with *E. huxleyi* and the physiological results from the monospecific study, microcosm and mesocosm tests with mixed phytoplankton species or natural phytoplankton assemblages, the present work suggests that a novel phenolic compound metabolism pathway, involving β-oxidation and the Krebs cycle, was enhanced by OA. While there are controversial findings on the effects of OA on mitochondrial respiration^8^,^9^,^10^, rising pCO_2_ and decreasing pH in seawater perturb the cytoplasmic acid–base balance of phytoplankton^11^, so that extra energy would be required to maintain the cell's homoeostasis or positive H^+^ efflux^12^. Enhanced photorespiratory carbon loss in high CO_2_ grown cells^9^,^13^ consumes additional energy for photoprotection. Consequently, an extra energy requirement for maintaining homoeostasis when phytoplankton cells are perturbed by changed seawater chemistry can be expected. In this study, we demonstrated that β-oxidation and the Krebs cycle were enhanced under OA and could thus meet any extra energetic demand to allow phytoplankton to tolerate acidic stress. For coccolithophores, increased phenolic compounds may not only reflect a way to endure with any extra energetic demand under OA but may also act as repellents to protect them from grazers as the cells calcify less under OA^1^.

It is a well-known phenomenon in higher plants that they increase the production of phenolic compounds to deter grazers. Our finding that phytoplankton increased the production of these compounds under high CO_2_ thus has implications for grazers, though the mechanism by which phytoplankton species or assemblages upregulate phenolic biosynthesis in response to increased pCO_2_ is not immediately clear. In contrast to our results, a recent study showed decreased content of these compounds in seagrasses exposed to high CO_2_/low pH condition near a natural CO_2_ vent^14^. OA has been shown to significantly alter fatty acid content and composition in diatoms^6^. The increased cellular phenolic compounds (shown in this work, Fig. 2a,c), also linked to fatty acid metabolism via β-oxidation, would further decrease the nutritional value of these organisms. Since phenolic compounds are highly toxic and are found in marine systems^15^,^16^, an increase in their content in primary producers would undoubtedly lead to significant consequences for food webs and carbon cycles. In addition, as phenolics are known to possess antimicrobial properties^17^, biogeochemical cycles in the oceans may be affected as well.

The present work demonstrated that accumulation of phenolic compounds increased in phytoplankton under OA and that they were transferred to higher trophic levels (zooplankton). Consequently, accumulation of phenolic compounds in seafood could be a factor that affects the quality of seafood^18^ when the organisms are exposed to OA, which was recently shown to affect the taste of shrimps^19^. Different taxa are known to show differential sensitivities to OA^20^, which can be altered or amplified under other forcing from ocean changes, such as warming^21^ and ultraviolet-B irradiance^22^. At the same time, phytoplankton species can exhibit evolutionary responses to OA^23^, and the changes in the profile of phenolic compounds remain unknown for long-term adaptation to OA. While altered biochemistry of the diatom *Thalassiosira pseudonana* grown under OA conditions can decrease egg production of a copepod^6^, exposure to phenol can also decrease egg, faecal pellet production and survival in copepods^24^,^25^ and toxic compounds can be transferred to higher trophic levels (this work). Therefore, an increase in the content of phenolic compounds in plankton could have far-reaching impacts on seafood qualitative and quantitative values and on species interactions as well as community structures, with consequences for ecosystem functioning and fishery industries.

## Methods

### Species and laboratory cultures

*E. huxleyi* (CCMP 1516), acquired from the Provasoli-Guillard National Center for Culture of Marine Phytoplankton (CCMP), was grown semi-continuously in high (HC, 1,000 μatm, pH_NBS_ 7.81) or low (LC, 395 μatm, pH_NBS_ 8.16) pCO_2_ (with the HC representing CO_2_ levels projected for the end of this century^26^) pre-equilibrated artificial seawater enriched with Aquil culture medium. The HC and LC cultures (triplicate independent cultures for each treatment) were maintained in exponential growth phase by continual dilution (every 24 h) for 20 generations before being used in the experiments, and the seawater carbonate system parameters were maintained at stable levels (daily variation in pH_NBS_ <0.06, seawater carbonate system parameters see Supplementary Note 1 and Supplementary Table 3) by using freshly prepared medium equilibrated with the target CO_2_ levels and by sustaining cell concentration within a range of 2.0–4.5 × 10^4^ cells per ml. The target CO_2_ levels of HC and LC medium were achieved by using a CO_2_ Enrichlor (CE-100B, Wuhan Ruihua Instrument & Equipment Ltd, China) and bubbling with ambient air, respectively. The cells were grown under a photon flux density of 100 μmol photons per m^2^ s^−1^ (12:12 light: dark cycle) in a plant growth chamber (GXZ, Ruihua, Wuhan, China) at 20 °C.

### Proteomics analysis

After acclimation for 20 generations, HC- and LC-grown *E. huxleyi* cells were collected for protein extractions, and then analysed for proteomics by applying a two-dimensional electrophoresis (2-DE) gel and MALDI-TOF-TOF mass spectrometry (MS) approach^27^ to identify the differentially expressed proteins between HC and LC treatments.

Specifically, after the acclimation of *E. huxleyi* cultures at the relevant treatments for 20 generations, 3 L samples were collected onto PC filters (Millipore, pore size 0.4 μm), re-suspended in pre-prepared medium equilibrated with the target CO_2_ level (pH_NBS_ 7.81 and 8.16 for the HC- and LC-grown cells, respectively) and then re-collected by centrifugation at 10,000 × *g* for 30 min at 4 °C for protein extraction. The cell pellets were rinsed twice with precooled sterilized seawater to avoid any carry-over of culture medium and external proteins. Trizol reagent (1 ml) was added to the cell pellet followed by sonication (a total of 2 min with short pulses of 3–5 s) on ice. Cell lysis was confirmed using light microscopy. Subsequently, 200 μl of chloroform was added to the cell lysate before shaking vigorously for 15 s. The mixture was allowed to stand for 5 min at room temperature before being centrifuged at 12,000 × *g* for 15 min at 4 °C. The top pale yellow or colourless layer was removed, and then 300 μl of ethanol was added to re-suspend the reddish bottom layer, and the mixture centrifuged at 2,000 × *g* for 5 min at 4 °C. The supernatant was then transferred to a new tube, and 2 ml of isopropanol was added. The mixture was allowed to stand for at least 1 h for precipitation of proteins at −20 °C. It was then centrifuged at 14,000 × *g* for 30 min at 4 °C. Subsequently, the pellet was washed with 95% ethanol before being air-dried. To solubilize the protein pellet, 30 μl of rehydration buffer (7 M urea, 2 M thiourea, 4% w/v 3-[(3-cholamidopropyl) dimethyl-ammonio]-1-propanesulfonate (CHAPS), 1% dithiothreitol (DTT) and 0.5% v/v immobilized pH gradient (IPG)) were added. The resulting solution was centrifuged at 20,000 × *g* for 30 min at 4 °C and the supernatant was collected for 2-DE analysis. The protein content was quantified using a 2-D Quant kit (GE Healthcare, San Francisco, USA).

Exactly 100 μg of protein sample (duplicates for each CO_2_ treatment) was mixed with a rehydration buffer (7 M urea, 2 M thiourea, 4% w/v 3-[(3-cholamidopropyl) dimethyl-ammonio]-1-propanesulfonate, 1% DTT, and 0.5% v/v IPG) before being loaded onto IPG strips with a linear pH gradient of 4–7 (Immobiline Drystrip, pH 4–7, GE Healthcare Life Science, Piscataway, USA). The sample was subjected to isoelectric focusing using an IPGphor III system with 24 cm IPG strips in the following manner: 6 h at 40 V (active rehydration), 6 h at 100 V; 0.5 h at 500 V; 1 h at 1,000 V; 1 h at 2,000 V; 1.5 h at 10,000 V; and 60,000 Vh at 10,000 V. The minimal Vh applied was at least 60,000 units. Subsequently, the immobilized pH gradient strips were equilibrated for 15 min in 10 ml of equilibration buffer containing 6 M urea, 2% SDS, 50 mM Tris-Cl (pH 8.8), 30% glycerol and 1% DTT, followed by equilibration for 15 min in alkylation buffer containing 6 M urea, 2% SDS, 50 mM Tris-Cl (pH 8.8), 30% glycerol and 2.5% iodoacetamide. Two-dimensional SDS–PAGE (2-DE) gels (12.5%) were run in an EttanDalt system (GE Healthcare) at 1 w per gel for 30 min and then at 15 w per gel for 6 h. The 2-DE gels were visualized using Coomassie Blue staining and digitized using a gel documentation system on a GS-670 Imaging Densitometer from Bio-Rad (USA) with 2-DE electrophoretogram-matching software. Image analysis was performed using DeCyder version 7.0 software (GE Healthcare) following the manufacturer's instructions.

MS analyses were conducted using an AB SCIEX MALDI TOF-TOF 5800 Analyser (AB SCIEX, Shanghai, China) equipped with a neodymium: yttrium-aluminum-garnet laser (laser wavelength was 349 nm), in reflection positive-ion mode. Protein identification was conducted according to the previously described method^27^. Briefly, the MS and MS/MS spectra of each protein spot obtained from MALDI-TOF-TOF MS were searched against the NCBI non-redundant protein database using the BLASTX algorithm. If the total ion score confidence interval was above 95% and the *E* value was below e^−20^ at the amino acid sequence level, the sequence similarities were considered to be significant. The details of NCBI ID number, theoretical pI value, theoretical molecular weight, protein score, protein score confidence interval %, as well as the average relative change are listed in Supplementary Table 1.

### Microcosm test

Microcosms of 30 L (water-jacketed for temperature control) were run from December 2014 to January 2015 at Wuyuan Bay (Xiamen, China) on the mesocosm facility platform (24.52°N, 117.18°N. Surface seawater (0–1 m) was collected at midday, filtered (200 μm) to remove large grazers and dispensed into the microcosms (triplicate microcosms were run for each CO_2_ treatment). The microcosms were made of polymethyl methacrylate, which allowed 91% photosynthetically active radiation, 63% ultraviolet-A (315–400 nm) and 6% ultraviolet-B (280–315 nm) transmissions under the incident solar radiation. The temperature within the microcosms was controlled to the sea surface temperature (13.0–15.0 °C) by circulating *in situ* seawater through the jacket. The seawater carbonate system in the microcosms was maintained stable by aerating with air of high (HC, 1,000 μatm) or low (LC, 395 μatm) pCO_2_ (see CO_2_ manipulation method in ref. 9). Samples of 100 ml from each microcosm were collected for the determination of phenolic compounds on day 5, while the microcosms were exposed to solar irradiance and run until the end of exponential growth phase of the phytoplankton growth (9 days).

### Mesocosm test

The Facility for Ocean Acidification Impacts Study of XMU consists of eight independently operated mesocosm units, located at 24.52°N, 117.18°N, Wuyuan Bay (Xiamen, China), of which three HC (HC, 1,000 μatm CO_2_) and three LC (LC, 395 μatm CO_2_) units were randomly chosen (for details see in Supplementary Note 2). Four species of phytoplankton, *Phaeodactylum tricornutum* (CCMA 106), *T. weissflogii* (CCMP 1335), *E. huxleyi* (CS-369) and *Gephyrocapsa oceanica* (NIES-1318) (details about the species are given in Supplementary Note 2) were then inoculated into each mesocosm at equivalent chlorophyll *a* concentrations to give a total final concentration of 50 cells per L on 15 June 2013 (day 0). The pCO_2_ in the mesocosms was controlled by bubbling air of high (HC, 1,000 μatm) or low (LC, 395 μatm) pCO_2_ (details for pCO_2_ manipulation are provided in the Supplementary Note 2).

### Estimation of phenolic compounds

Phenolic content was determined according to ref. 28. Briefly, the cell pellets of phytoplankton or ∼300 zooplankton individuals were placed in 2.5 ml of 95% ethanol for a period of 48 h at 47 °C. The cells were sonicated and the supernatant was separated by centrifugation (4,500 × *g*) for 10 min, 1.0 ml of which was transferred to glass test tubes along with 1.0 ml 95% ethanol, 5.0 ml distilled water and 0.5 ml of 50% Folin–Ciocalteu reagent (Sigma Chemical, USA). The solution was allowed to react for 5 min, then 1.0 ml of 5% Na_2_CO_3_ was added, and the mixture was vortexed and placed in the darkness for 1 h. Absorbance was determined with a scanning spectrophotometer (DU800, Beckman, Fullerton, CA, USA) at 725 nm and plotted against a standard curve obtained from gallic acid.

### Measurement of respiration

Mitochondrial respiration rates of laboratory cultures were determined by a Clark-type oxygen electrode in darkness. The respiratory carbon loss in phytoplankton assemblages from the mesocosms over 12 h was calculated as the difference in the amount of fixed carbon, using ^14^C tracer methods, between the two time spans (carbon fixation 12 h–carbon fixation 24 h; for details, see the Supplementary Note 3).

### Feeding experiments

Zooplankton individuals were obtained at night through horizontal hauling with a medium plankton net (mesh diameter, 112 μm) from surface water in Wuyuan Bay. Collected zooplankton samples were nursed in culture dishes (400 ml) in *in situ* seawater pre-equilibrated with target high (1,000 μatm) or low pCO_2_ (395 μatm) levels for 12 h before using them. Zooplankton in each culture under HC or LC treatment (three independent replicates for each CO_2_ treatment) were fed with either HC- or LC-grown phytoplankton cells collected from the microcosms or mesocosms at about 15 μg chlorophyll *a* per L every 12 h. The cultures were maintained under solar radiation by covering with five neutral density screens (providing 6% of incident solar radiation). After the feeding procedure (24 h), zooplankton samples were collected for measurements of phenolic content and sub-samples were collected for microscopic enumeration.

## Additional information

**How to cite this article:** Jin, P. *et al*. Ocean acidification increases the accumulation of toxic phenolic compounds across trophic levels. *Nat. Commun*. 6:8714 doi: 10.1038/ncomms9714 (2015).

## Supplementary Material

Supplementary Information Supplementary Figures 1-2, Supplementary Tables 1-3, Supplementary Notes 1-4 and Supplementary References
