# Supplementary material for: Ocean acidification increases the accumulation of toxic phenolic compounds across trophic levels
Source: Nat Commun. 2015 Oct 27;6:8714. doi: 10.1038/ncomms9714 (PMC4640080; doi:10.1038/ncomms9714)
Supplement: Supplementary Information — Supplementary Figures 1-2, Supplementary Tables 1-3, Supplementary Notes 1-4 and Supplementary References [file ncomms9714-s1.pdf]

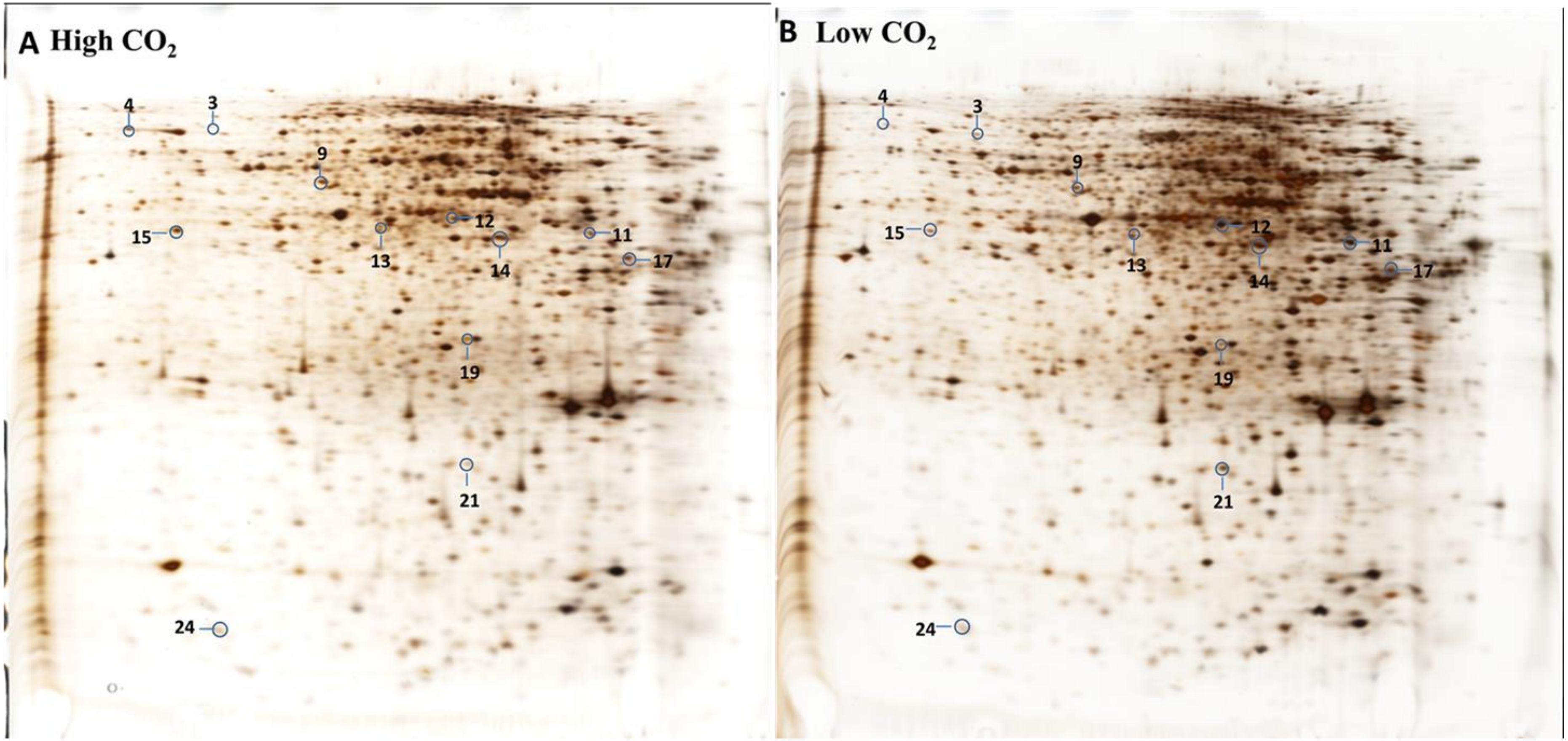

Supplementary Figure 1: 2D-gel profiles of the proteins of *E. huxleyi* grown under the high (HC, 1000 µatm, pH_{NBS} 7.81) or low (LC, 395 µatm, pH_{NBS} 8.16) pCO_2 conditions for 20 generations. The numbered spots are those showing statistically significant alterations (greater than 2-fold) in abundance.

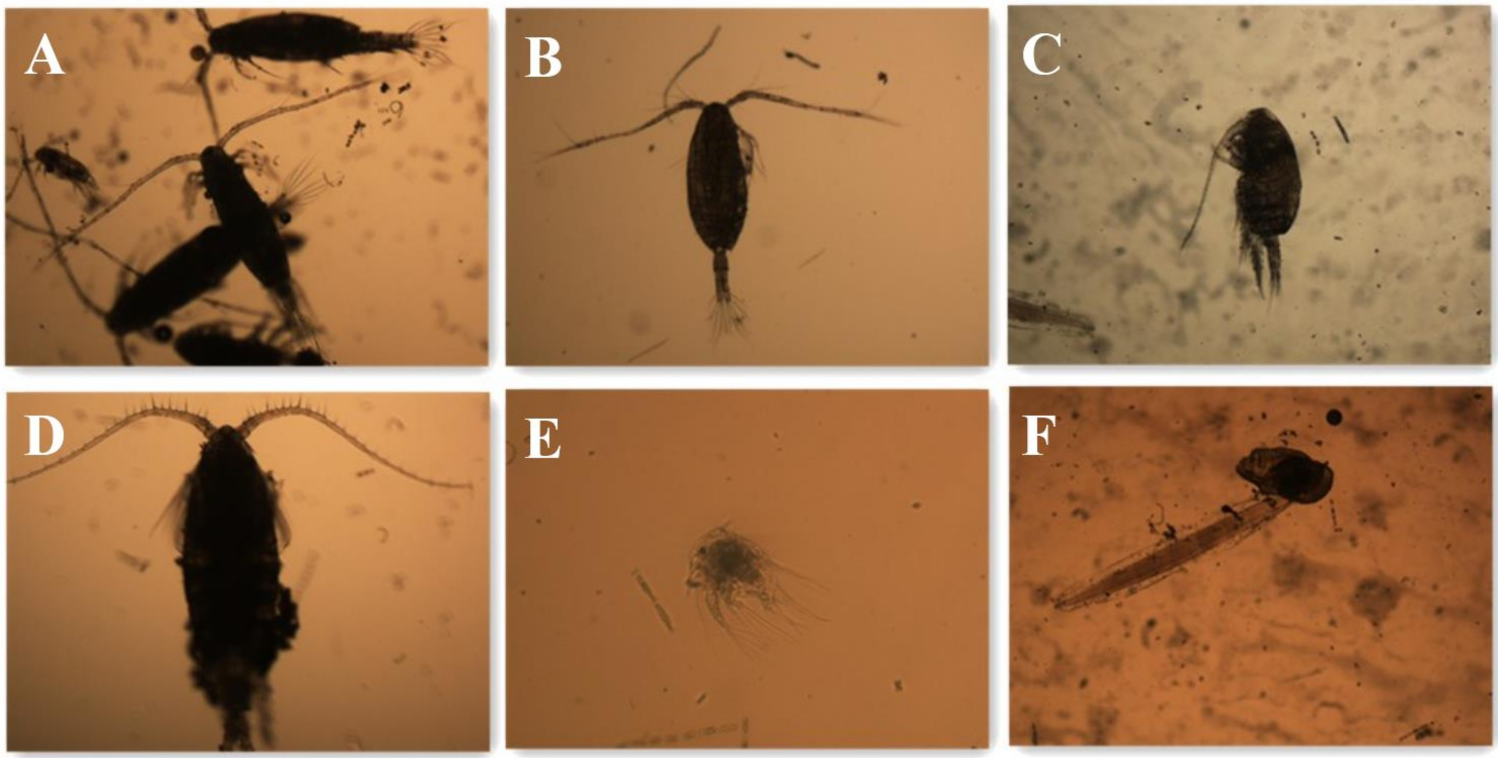

Supplementary Figure 2: Microscope images of zooplankton species in the microcosm test. The natural zooplankton assemblages were dominated by calanoid copepods (abundance up to ~95%) (A, B, C, D). A, B: *Acartia pacifica* (abundance up to ~60%, dominant species); C: *Paracalanus* sp. (abundance up to ~20%); D: *Schmackeria* sp. (abundance up to ~15%); E: Cirriped larva and F: Ascidian larva 5% in total.

Supplementary Table 1 Various proteins, that showed greater than 2-fold alterations in abundance, in *E. huxleyi* cells grown under high (HC, 1000 µatm, pH_{NBS} 7.81) or low (LC, 395 µatm, pH_{NBS} 8.16) pCO_2 levels for 20 generations.

Spot Id.	Protein identity	GI number	Protein score C.I. (%)	Total Ion C. I. %	Protein score (peptides)	MW/pI	Fold change		Function
							High CO_2	Low CO_2	
3	Propionyl CoA synthase	239994558	100	100	357(14)	69708.5/5.51	2.33	1.00	β-oxidation
4	Serine protein kinase	239995429	100	99.946	177(15)	74347.3/5.31	2.82	1.00	Protein kinase, signal transduction
9	Hypothetical protein AmacA_2	223994739	100	100	805(22)	51069.6/5.61	2.01	1.00	Unknown
11	Hypothetical protein MDMS009_211	254489880	100	100	440(11)	447891.1/4.87	1.00	4.34	Unknown
12	Methane/ phenol/ toluene hydroxylase	148260382	100	100	238(5)	39315.7/5.76	3.40	1.00	Phenol biodegradation
14	Chloroplast glyceraldehyde-3-phosphate dehydrogenase	77024139	100	100	336(7)	44096.1/5.2	2.93	1.00	Glycolysis
15	Conserved hypothetical protein (bacterium S5)	288797257	100	99.996	166(7)	21306.1/4.87	2.50	1.00	Unknown
17	Enoyl-CoA hydratase	83955054	99.996	98.89	115(8)	28178.9/5.51	3.82	1.00	β-oxidation

19	Adenylate kinase	239993306	100	100	600(16)	23693/4.99	2.12	1.00	ATP synthesis
21	TRAP-T family protein transporter periplasmic binding protein	83943788	100	100	811(17)	39967.7/4.56	3.04	1.00	Substrate-binding protein (SBP)-dependent secondary transporters
24	Nucleoside diphosphate kinase	114765301	100	100	352(6)	15293.7/4.93	1.00	2.10	Catalyze the transfer of a phosphate from a NTP to NDP

Supplementary Table 2 Significance levels of the differences in different physiological endpoints between HC- and LC-treatment in the laboratory, microcosm and mesocosm experiments. The interactive effects of CO_2 treatment and replicate were statistically analysed using one- or two-way ANOVA. "N" and "n" represents the number of replicates, and the number of observations per replicate for laboratory cultures, microcosms or mesocosms, respectively. Letters in bold indicate significance at $p < 0.05$ level.

	Endpoints	N	n	Total	Model	CO_2 treatment	Replicate	Interaction
Laboratory	pH	3	2	6	**$F_{5,6} = 116.74$, $p < 0.0001$**	**$F_{1,6} = 580.26$, $p < 0.001$**	$F_{2,6} = 0.96$, $p = 0.43$	$F_{2,6} = 0.75$, $p = 0.51$
	DIC	3	2	6	$F_{5,6} = 1.55$, $p = 0.30$	$F_{1,6} = 4.52$, $p = 0.08$	$F_{2,6} = 0.92$, $p = 0.45$	$F_{2,6} = 0.69$, $p = 0.54$
	TA	3	2	6	$F_{5,6} = 0.70$, $p = 0.64$	$F_{1,6} = 0.00$, $p = 0.99$	$F_{2,6} = 1.00$, $p = 0.42$	$F_{2,6} = 0.76$, $p = 0.51$
	HCO_3^-	3	2	6	$F_{5,6} = 3.16$, $p = 0.097$	$F_{1,6} = 12.90$, $p = 0.01$	$F_{2,6} = 0.84$, $p = 0.48$	$F_{2,6} = 0.61$, $p = 0.57$
	CO_3^{2-}	3	2	6	**$F_{5,6} = 18.06$, $p = 0.0015$**	**$F_{1,6} = 85.18$, $p < 0.001$**	$F_{2,6} = 1.38$, $p = 0.32$	$F_{2,6} = 1.17$, $p = 0.37$
	Phenol in phytoplankton	3	1	3		**$F_{1,3} = 119.53$, $p < 0.001$**		
	Respiration	3	1	3		**$F_{1,3} = 532.66$, $p < 0.001$**		
Microcosm	pH	3	1	3		**$F_{1,3} = 1210$, $p < 0.001$**		
	DIC	3	1	3		$F_{1,3} = 2.25$, $p = 0.21$		
	TA	3	1	3		$F_{1,3} = 2.52$, $p = 0.19$		
	HCO_3^-	3	1	3		**$F_{1,3} = 11.74$, $p = 0.027$**		
	CO_3^{2-}	3	1	3		**$F_{1,3} = 417$, $p < 0.001$**		
	Phenol in phytoplankton	3	2	6	**$F_{5,6} = 4.28$, $p = 0.048$**	**$F_{1,6} = 18.84$, $p = 0.005$**	$F_{2,6} = 0.74$, $p = 0.52$	$F_{2,6} = 0.05$, $p = 0.95$
	Phenol in zooplankton	3	2	6	**$F_{5,6} = 7.19$, $p = 0.016$**	**$F_{1,6} = 33.24$, $p = 0.001$**	$F_{2,6} = 0.95$, $p = 0.44$	$F_{2,6} = 0.41$, $p = 0.68$
Mesocosm	pH	3	1	3		**$F_{1,3} = 456$, $p < 0.001$**		
	DIC	3	1	3		$F_{1,3} = 0.19$, $p = 0.69$		
	TA	3	1	3		$F_{1,3} = 4.17$, $p = 0.11$		
	HCO_3^-	3	1	3		$F_{1,3} = 0.53$, $p = 0.51$		
	CO_3^{2-}	3	1	3		**$F_{1,3} = 54$, $p = 0.002$**		
	Phenol in phytoplankton	3	3	9	**$F_{5,12} = 10.91$, $p = 0.0004$**	**$F_{1,12} = 54.48$, $p < 0.001$**	$F_{2,12} = 0.02$, $p = 0.98$	$F_{2,12} = 0.02$, $p = 0.98$
	Phenol in zooplankton	3	2	6	**$F_{5,6} = 8.15$, $p = 0.012$**	**$F_{1,6} = 29.46$, $p = 0.002$**	$F_{2,6} = 5.33$, $p = 0.05$	$F_{2,6} = 0.32$, $p = 0.74$
	Respiration	3	1	3		$F_{1,3} = 2.78$, $p = 0.171$		

Supplementary Table 3. Parameters of the seawater carbonate system under the high (1000 µatm, HC) and low (395 µatm, LC) pCO_2 levels in the laboratory cultures (N = 3), microcosm (N = 3) and mesocosm (N = 3) tests. Carbonate chemistry parameters in the mesocosms represent those before the measurements (with Chl *a* concentration < 5 µg L^{-1}). Measurements and estimation of the parameters are described in the Supplementary Note 1. "N" represents the number of replicates for laboratory cultures, microcosms or mesocosms, respectively.

	Treatment	pCO_2 (µatm)	pH_{NBS}	DIC (µmol kg^{-1})	HCO_3^- (µmol kg^{-1})	CO_3^{2-} (µmol kg^{-1})	Total alkalinity (µmol kg^{-1})
Laboratory	HC	1000	7.81±0.02	2086.4±100.3	1960.9±91.7	93.1±8.7	2189.3±110.8
	LC	395	8.16±0.03	1933.2±136.6	1735.4±112.5	185.0±24.2	2190.0±165.3
Microcosm	HC	1000	7.80±0.02	2028.6±82.0	1908.2±75.2	88.1±6.9	2125.5±90.4
	LC	395	8.17±0.01	1953.4±28.2	1752.6±23.2	188.0±5.0	2214.0±34.1
Mesocosm	HC	1000	7.77±0.01	1903.8±26.0	1793.7±24.0	77.8±2.1	1988.1±28.6
	LC	395	8.16±0.03	1941.1±146.7	1741.9±121.1	186.3±25.6	2199.5±177.1

Supplementary Note 1: Carbonate system determination

The pH in the cultures was measured daily with a pH meter (Benchtop pH510, OAKTON) that was calibrated with National Bureau of Standards (NBS) buffer solution (Hanna). The parameters of the seawater carbonate system (Supplementary Table 3) were calculated from pH and pCO_2 or measured values of DIC using CO2SYS software[1], and cross-checked with DIC or pCO_2, using the equilibrium constants of K_1 and K_2 for carbonic acid dissociation of Roy et al. (1993)[2]. Under the elevated CO_2 condition, the carbonate system in the high pCO_2 seawater differed significantly from that of the control (Supplementary Table 3, Statistical details in Supplementary Table 2).

Supplementary Note 2: Mesocosm setup

Each mesocosm was constructed from a cylindrical transparent thermoplastic polyurethane (TPU) bag with a dome (made of the same TPU) to reduce the contamination risk and prevent dilution from rainfall. Each bag was 3 m deep and 1.5 m wide. The mesocosms were filled simultaneously with filtered (0.01 μm) *in-situ* seawater within 24 hrs. The inoculated phytoplankton strain of *Phaeodactylum tricornutum* (CCMA 106) was isolated from the South China Sea (SCS) in 2004 and obtained from the Center for Collections of Marine Bacteria and Phytoplankton (CCMBP) of the State Key Laboratory of Marine Environmental Science (Xiamen University), while *Thalassiosira weissflogii* (CCMP 102) was obtained from CCMP (the Provasoli-Guillard National Center for Culture of Marine Phytoplankton) and maintained axenically in CCMBP. The coccolithophorid *Emiliania huxleyi* (CS-369) was obtained from the Commonwealth Scientific and Industrial Research Organization (CSIRO, Australia), while the coccolithophorid *Gephyrocapsa oceanica* (NIES-1318) was originally isolated from the East China Sea and obtained from the National Institute for Environmental Studies in Japan. The pCO_2 in the mesocosms was controlled by bubbling with air of high (HC, 1000 μatm) or low (LC, 395 μatm) pCO_2. Specifically, the HC condition was achieved by using a CO_2 Enrichlor (CE-100B, Wuhan Ruihua Instrument & Equipment Ltd, China). The air with target CO_2 concentrations was delivered at the bag's bottom at

a flow rate of approximately 5 L min^{-1} and dispersed by an air stone. The bubbling was continuous in order to compensate for the inorganic carbon draw-down due to photosynthesis.

Supplementary Note 3: Measurement of respiration

In the laboratory cultures, cells were harvested by filtering, and then re-suspended in Tris-buffered medium (pH 8.17 and 7.82 for HC and LC acclimated cells, respectively). Respiratory O_2 uptake was measured using a Clark-type oxygen electrode (5300A, Yellow Springs Instruments, USA) in darkness at a constant temperature of 20 ± 0.1^{o}C, which was controlled by a recirculating cooler (CTP-3000, Eyela, Tokyo, Japan).

In the mesocosms, all of the tubes containing phytoplankton samples inoculated with ^{14}C were placed into a water bath through *in-situ* seawater which was circulated to control the temperature (28.5-29.5 ^{o}C), and covered with one layer of neutral density screen to reduce the PAR level to 55% of the incident sunlight, which reflects mean levels of sunlight within the mesocosms. After 12 h and 24 h, respectively, the cells were filtered onto a Whatman GF⁄F glass fiber filter (25 mm), then immediately frozen and stored at -20 ^{o}C for later measurements. The frozen filter was put in a 20 mL scintillation vial and exposed to HCl fumes overnight and dried (60 ^{o}C, 3 h) to remove the non-incorporated inorganic carbon[3]. Scintillation cocktail (5 mL) was then added to each vial and the radioactivity was counted with a liquid scintillation counter (LS 6500, Beckman Coulter, USA).

Supplementary Note 4: Species analysis in microcosms

For species analysis in microcosms, both HC and LC preconditioned phytoplankton samples were fixed with buffered formalin (final concentration of 0.4%) before feeding experiments. The dominant species were determined using an inverted microscope (IX51, OLYMPUS, Japan). We did not carry out detailed quantitative analyses here, but we have confirmed that the dominant species in HC microcosms did not differ from those of LC microcosms.

Supplementary References

1 Lewis, E. & Wallace, D. Program developed for CO_2 system calculations. *ORNL/CDIAC-*

105. Carbon Dioxide Information Analysis Center, Oak Ridge National Laboratory, US Department of Energy, Oak Ridge, Tennessee (1998).

2 Roy, R. N. *et al.* The dissociation constants of carbonic acid in seawater at salinities 5 to 45 and temperatures 0 to 45 ^{o}C. *Mar Chem* **44**, 249-267 (1993).

3 Gao, K. *et al.* Solar UV radiation drives CO_2 fixation in marine phytoplankton: A double-edged sword. *Plant Physiol* **144**, 54-59, doi: 10.1104/pp.107.098491 (2007).
